# Hidradenitis Suppurativa’s Impact on Social Activities: An Observational Study

**DOI:** 10.7759/cureus.25292

**Published:** 2022-05-24

**Authors:** Patrick O Perche, Rohan Singh, Aditi Senthilnathan, Steven R Feldman, Rita O Pichardo

**Affiliations:** 1 Dermatology, Wake Forest School of Medicine, Winston Salem, USA; 2 Dermatology, Pathology, and Social Sciences & Health Policy, Wake Forest School of Medicine, Winston Salem, USA

**Keywords:** body regions, painful nodules, hurley score, quality of life, recreational activities, social activities, hidradenitis suppurativa

## Abstract

Hidradenitis suppurativa (HS) can severely impact patients’ quality of life. However, its specific impact on participation in everyday social activities is not well characterized. We recruited a cohort of patients with HS to complete a survey assessing the degree of interference HS has on participation in social activities. Patients also completed the Brief Fear of Negative Evaluation (BFNE) to assess levels of social anxiety. The majority of patients in our cohort, regardless of Hurley Stage, reported interference with social activities, and patients with more painful nodules and greater body region involvement reported greater interference with social activities. BFNE scores were high in our cohort, and patients with higher BFNE scores reported greater interference in all social activities assessed. Both the physical and psychological effects of HS may interfere with patients’ participation in social activities, and efforts to treat both aspects may improve quality of life.

## Introduction

Hidradenitis suppurativa (HS) is a debilitating, inflammatory condition of apocrine-bearing skin, characterized by nodules, abscesses, fistulae, and sinus tract formation [1]. Multiple aspects of patients’ lives are negatively affected and patients frequently report feeling isolated [2,3]. There is limited information on how HS may impact patients’ participation in everyday social activities. We assessed how much HS impacted the ability of patients to participate in social activities and examined whether a greater propensity to feel social anxiety was associated with a greater impact on social activities.

## Materials and methods

We approached 153 patients (n= 123 mail, 30 clinics) diagnosed with HS (ICD-10 code: L 73.2) between June and September 2018 from the Atrium Health Wake Forest Baptist Department of Dermatology clinics after Institutional Review Board approval was obtained. A total of 67 surveys were received (mail, n=40; clinic, n=27) and analyzed. Disease severity was assessed via a validated self-assessment tool using the Hurley Staging system [4]. Respondents reported the number of painful nodules and the number of body regions involved. A series of questions were asked on the extent to which HS interfered (a lot, a little, none) with social activities. We assessed whether aspects of objective disease severity (Hurley score, number of body regions affected, and number of painful nodules) correlate with interference with social activities. Respondents also completed the Brief Fear of Negative Evaluation (BFNE), a validated questionnaire that assesses the tendency toward feeling social anxiety [5]. The BFNE consists of 12 questions, each graded on a 1-5 Likert Scale (1 = not at all like me, 2 = slightly like me, 3 = moderately like me, 4 = very like me, 5 = extremely like me), with a range of 12 to 60; higher scores indicate greater tendency to experience social anxiety [5]. Respondent scores were grouped into high BFNE (≥ 31) and low BFNE (≤ 30) based on the median cut-off. We assessed whether higher BFNE scores were associated with greater interference with social activities. Data were analyzed using SAS Software 9.4. Differences in percent of respondents were analyzed using Chi-squared and Fisher exact analysis.

## Results

The demographics of respondents (mean age: 39, 90% female, 57% African American [AA]) were comparable to non-responders (mean age: 36, 80% female, 38% AA). Respondents had an average BMI of 35.7, 56% had a family history of HS, 28% were current smokers, 22% had Hurley stage 1, 35% had Hurley stage 2, and 43% had Hurley stage 3 disease severity; average disease duration was 13.8 years. Most respondents reported HS interfered with participation in sports or recreational activities (69.4%), going out socially or to a special event (66.1%), hobbies (60.0%), going to parties (54.0%), going out in public (52.3%), and being with family members (41.3%; Table 1). More respondents with >1 body region with HS reported their disease impacted participation in sports or recreational activities, going out socially or to special events, participation in hobbies, and going to parties, a lot, compared to respondents with one body region involvement (50.8%, 32.3%, 32.3%, 33.3% vs. 0%; p=0.008, 0.002, 0.02, 0.04, respectively; Table 1). Moreover, more patients with >5 painful nodules reported their HS impacted participation in sports or recreational activities and going out socially or to a special event, a lot, compared to patients with ≤5 painful nodules (59.3%, 37.9% vs. 36.1%, 24.3%; p=0.04, 0.006, respectively; Table 1). Mean BFNE was 31.5 (median 31). Higher BFNE/greater tendency toward feeling social anxiety was associated with greater interference in activities for all activities assessed (Table 1).

**Table 1:** Percent of respondents reporting hidradenitis suppurativa (HS) interfered a lot and a little in various social activities as a function of Hurley score, number of body regions affected, and number of painful nodules; Percent of respondents with high and low Brief Fear of Negative Evaluation (BFNE) scores reporting HS interfered a lot and a little in various social activities. p<0.05*, p<0.01**

Social Activity	Degree of Interference	Disease Severity per Hurley Score				Number of Body Regions with HS		Number of Painful Nodules		High BFNE (≥31)		Low BFNE (≤30)
		1	2	3	All 3	1	>1	≤5	>5	Mean BFNE=38.6	Mean BFNE=24.2	
		n=17	n=21	n=27	n=65	n=6	n=60	n=37	n=28	n=32	n=31	
Participating in sports or recreational activities	Lot	37.5%	47.6%	52.0%	46.8%	0%**	50.8%**	36.1%*	59.3%*	62.5%**	29.0%**	
	Little	31.3%	14.3%	24.0%	22.6%	16.6**	22.0%**	19.4%*	25.9%*	18.8%	25.8%	
Going out socially or to a special event	Lot	27.8%	23.8%	34.6%	29.2%	0% **	32.3%**	24.3%**	37.9%**	43.8%*	16.1%*	
	Little	22.2%	33.3%	50.0%	36.9%	0%**	38.7%**	24.3%**	48.3%**	31.2%	45.2%	
Doing hobbies	Lot	16.6%	28.6%	42.3%	30.8%	0%*	32.3%*	29.7%	31.0%	43.8%*	16.1%*	
	Little	33.3%	33.3%	23.1%	29.2%	0%*	30.6%*	16.2%	37.9%	28.1%	47.6%	
Going to parties	Lot	17.6%	28.6%	40.0%	30.2%	0%*	33.3%*	27.0%	37.0%	50.0%**	12.9%**	
	Little	29.4%	33.3%	28.0%	23.8%	0%*	25.0%*	18.9%	32.6%	12.5%*	35.5%*	
Going out in public	Lot	17.6%	19.0%	25.9%	21.5%	0%	24.2%	18.9%	27.6%	40.6%**	6.5%**	
	Little	23.5%	28.6%	37.0%	30.8%	16.6%	32.3%	27.0%	37.9%	21.9%	41.9%	
Being with family members	Lot	5.9%,	9.5%	24.0%	14.3%	0%	16.6%	10.8%	22.2%	25.0%*	6.5%*	
	Little	23.5%	33.3%	24.0%	27.0%	0%	28.3%	21.6%	33.3%	34.4%	19.4%	

## Discussion

The majority of patients in our cohort, regardless of Hurley stage, reported HS interfered with their social activities, however, patients with more painful nodules and body regions affected reported a greater impact on their social activities. Moreover, subjects with a greater tendency toward social anxiety, as measured by BFNE, had greater interference in all social activities.

Participation in physical and social activities may improve patients’ disease severity and well-being, respectively, and lack thereof may worsen disease outcomes and contribute to feelings of isolation [6,7]. Social anxiety may be a contributing factor to HS patients’ degree of isolation. Currently, there are limited data on HS’s effect on participation in social activities. Quality of life improvement is needed for patients with this physically and psychologically debilitating disease [8]. Interventions, such as social support groups, may help improve the quality of life of patients with HS, who are already prone to isolation, particularly in patients with greater body region involvement and in patients with a tendency toward social anxiety [9]. Patients with a greater tendency toward feeling social anxiety may be in the greatest need of such support.

Limitations of our study include a small sample size recruited from a single academic center although our effect size was large enough to detect differences. Our non-response rate was high; however, the demographics of responders and non-responders were similar. We did not assess for reasons why HS interfered with various social activities, and while we did not assess causality, interventions to reduce isolation among patients with HS may be helpful to improve their quality of life.

## Conclusions

HS is a debilitating disease and patients have reported feelings of isolation in past studies. In our cohort, subjects reported their HS interfered with everyday social activities. These included participating in sports, going out socially or to a special event, participating in hobbies, going to parties, going out in public, or being with family. Moreover, subjects with a greater tendency to feel social anxiety reported greater interference in social activities, as compared to subjects with a lower tendency to feel social anxiety. The physical and psychological effects of HS impair quality of life. Holistic management of patients with HS may help decrease the disease burden.
